# Acrylonitrile Butadiene Styrene/Thermoplastic Polyurethane Blends for Material Extrusion Three-Dimensional Printing: Effects of Blend Composition on Printability and Properties

**DOI:** 10.1021/acsomega.3c06595

**Published:** 2023-11-17

**Authors:** Boonlom Thavornyutikarn, Chuanchom Aumnate, Wasana Kosorn, Nutdanai Nampichai, Wanida Janvikul

**Affiliations:** † National Metal and Materials Technology Center, National Science and Technology Development Agency, Pathum Thani 12120, Thailand; ‡ Metallurgy and Materials Science Research Institute, Chulalongkorn University, Bangkok 10330, Thailand

## Abstract

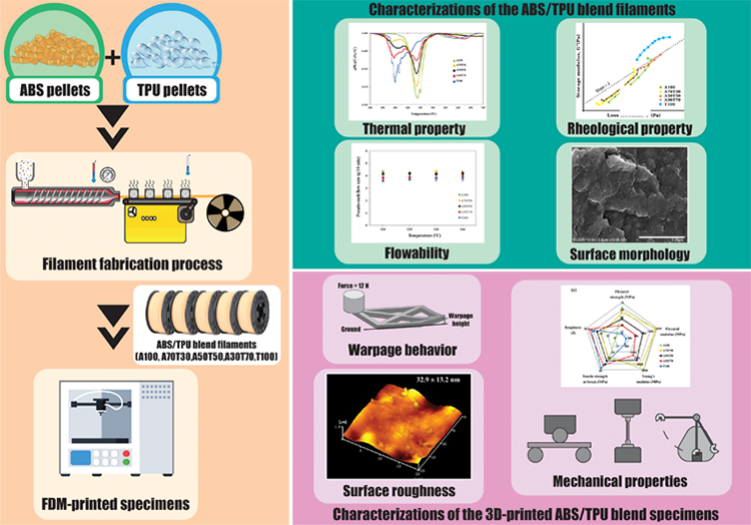

Blend filaments of acrylonitrile butadiene styrene (ABS) and thermoplastic polyurethane (TPU) were prepared at different weight ratios, i.e., 100:0, 70:30, 50:50, 30:70, and 0:100, for FDM printing; the prepared filaments, with an average diameter of 2.77 ± 0.19 mm, were encoded as A100, A70T30, A50T50, A30T70, and T100, respectively. The properties and printability of the filaments were thoroughly investigated. The blend composition, as well as the printing parameters, were optimized to obtain the FDM-printed objects with a well-defined surface structure and minimized warpages. The glass transition temperatures of ABS and TPU in the blends were not much altered from those of the parent filaments, whereas the thermal degradation characteristics of the blend filaments still fell between those of the neat filaments. The fractured surfaces of the filaments, observed by SEM, appeared smoother when higher amounts of TPU integrated; the smoothest surface of the ABS-based filament was found in A30T70, indicating the well-compatible blend characteristic. This was also confirmed by its rheological behavior examined by a parallel plate rheometer at 225 °C. Not only was the printability of the filaments improved, but also the warpages of the 3D-printed specimens were decreased when increasing amount of TPU was incorporated into the filaments. Among the printed objects, the A30T70 specimen exhibited the evenest surface morphology with the lowest surface roughness value of 32.9 ± 13.2 nm and the most uniform and consistent linear printing structure when being fabricated at the nozzle temperature of 225 °C and the printing bed temperature of 60 °C. However, the incorporation of TPU into the filaments markedly cut down both strength and modulus values of the fabricated materials up to about half but assisted the printed articles to absorb more energy, demonstrating that this polymer served as a good and effective toughener for ABS.

## 1 Introduction

Additive manufacturing (AM), generally referred to as three-dimensional (3D) printing, is an emerging disruptive technology that is now used widespread in various applications, including automobile, aerospace, construction, and biomedical applications.^1−5^ It assists in improving the accuracy and reproducibility of custom-made products with geometric designs. Among the 3D printing techniques, such extrusion-based 3D printing as fused deposition modeling (FDM), is the most broadly employed because of its ease to operate and low cost.^6^ Despite their versatility and affordable cost, one of the major constraints for developing FDM-printed specimens with desirable properties is the limited availability of commercial FDM printable materials. Currently, the most extensively exploited filament materials for FDM are thermoplastic materials such as polylactic acid (PLA), acrylonitrile butadiene styrene (ABS), and polycarbonate (PC). There still exist a few types of polymer blend filaments being utilized nowadays.

Polymer blending is an efficient and cost-effective process to prepare polymeric materials with desired mechanical properties, as well as ease of processability and printability.^7−10^ The development of FDM filament feedstocks from new polymer blends for FDM has been of great interest, as they often offer enhanced material performances and particularly unique functionalities when compared to their corresponding homopolymers. For instance, the study of Geng et al.^7^ demonstrated that the blending of PLA and PC markedly improved the filament processability as well as FDM printability, with the resulting polymer blend filaments. Additionally, 3D printed PC/PLA specimens exhibited superior impact strength and toughness. Another study by Fekete et al.^9^ also reported that the addition of natural rubber (NR) (5–20 wt %) improved the ductility and toughness of NR-blended PLA filaments, compared to those of pure PLA filament.

ABS, an amorphous thermoplastic polymer, is commonly employed as one of the build materials in FDM studies, as it has a high tensile strength and excellent impact resistance. This engineering plastic has been extensively exploited in various applications.^11,12^ However, ABS is prone to thermal shrinkage and warpage during FDM printing due to its high manufacturing temperature and high coefficient of thermal expansion.^13,14^ Recently, attempts to combine ABS with thermoplastic polyurethane (TPU) have been explored for the development of high-performance FDM filaments/specimens^15−17^ as TPU possesses a relatively lower coefficient of thermal expansion and requires a relatively lower extrusion temperature, resulting in reduced internal stresses upon cooling.^18^ Moreover, this biocompatible material can be bonded with multiple thermoplastics.^19^ For instance, the study by Yin et al.^15^ revealed the good intermolecular diffusion between ABS and TPU layers of 3D specimens printed by FDM, leading to heightened mechanical properties and functions of the multimaterial products, compared with those of the 3D printed parent materials. Another investigation by De León et al.^17^ disclosed that the FDM-printed specimen from an ABS/TPU filament blended with 30 wt % TPU had good adhesion not only to the FDM platform but also between the printed layers and improved mechanical properties as a result of the strong hydrogen bonding formed between ABS and TPU in the blend.

A thorough understanding of the properties, including rheology behavior, of a newly developed polymer blend filament is essentially a prerequisite for FDM printing. Generally, the flow behavior of a polymer blend is rather complex and influenced by several factors such as miscibility, morphology, and interfacial adhesion.^20^ Many reviews have been published on the rheological studies on the melt state of the polymer blends in their linear viscoelastic regions to evaluate miscibility using the Han plot or the log–log plot of the storage modulus (*G*′) versus the loss modulus (*G*″). A slope of 2 in Han plots can be used as a criterion for the homogeneous phase behavior.^21−23^ Reinaldo et al.^23^ performed a rheological study on the acrylic and styrenic polymer blends in the linear viscoelastic region. The correlations of the rheological properties from the Han plots could be effectively used to confirm the miscibility observation in the blend system.

Up to now, the combinatorial uses of ABS and TPU blended materials containing more than 30 wt % TPU for development of high-performance FDM filaments have scarcely been reported. To do so, the rheological, mechanical, and thermal properties of the blend filaments have yet to be determined prior to usage. Hence, in this study, a melt-mixing method was employed for the preparation of a series of high-performance ABS/TPU blend filaments containing 0, 30, 50, 70, and 100 wt % TPU. The thermal, flowability, morphology, and rheological properties of the blends were comparatively examined and correlated to their processability and miscibility. Subsequently, the FDM printing parameters, particularly the printing temperature and printing bed temperatures, were optimized to obtain properly FDM-printed blend objects with minimized warpages. At last, the morphological and mechanical properties of the as-printed objects were thoroughly investigated.

## 2 Experimental Procedures

### 2.1 Materials

Acrylonitrile butadiene styrene (ABS) with a density of 1.05 g/cm^3^ and a melt flow index (MFI) of 20 g/10 min (220 °C) was purchased from IRPC Public Company Limited, Thailand. Thermoplastic polyurethane (TPU) with a density of 1.23 g/cm^3^ and a shore hardness of 65D was obtained from Able One Engineering Co. Ltd., Thailand. The mechanical properties of both commercial materials are listed in Table 1.

**Table 1:** Mechanical Properties of ABS and TPU are Indicated in the Technical Data Sheets Issued by the Manufacturers

	ABS	TPU
tensile strength (MPa)	52 (ASTM D638)	45 (DIN 53504)
flexural strength (MPa)	70 (ASTM D790)	
elongation at break (%)		300 (DIN 53504)

### 2.2 Extrusion of Polymer Blend Filaments

**Scheme 1:**
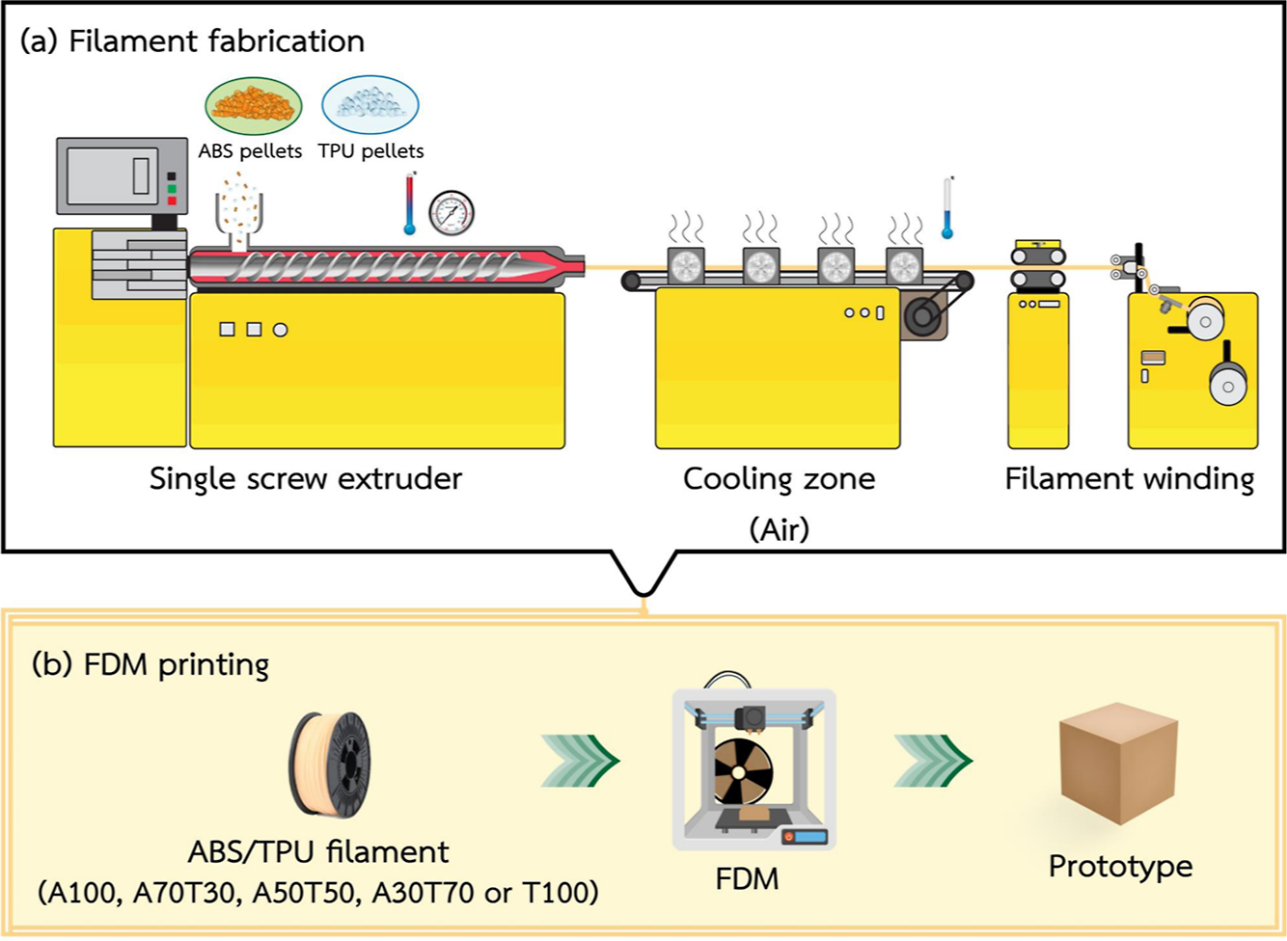
Schematic Illustration of (a) the Preparation of 3D Printing ABS/TPU Blend Filaments and (b) the FDM-Printing of 3D ABS/TPU Blend Specimens

The preparation of 3D printing ABS/TPU blend filaments is depicted in Scheme 1a. Individual ABS and TPU pellets were primarily dried in an oven overnight at 80 °C before being blended at various weight ratios, i.e., 100:0, 70:30, 50:50, 30:70, and 0:100 (encoded as A100, A70T30, A50T50, A30T70, and T100, respectively). Each blend was prepared by a melt-blending method using a single-screw extruder (HAAKE PolyLab OS RheoDrive7, Thermo Scientific, USA) having a screw diameter of 19 mm, a L/D ratio of 25, and a rod die with a diameter of 2.8 mm. The optimum barrel temperatures were set at 195, 205, and 210 °C, while the die temperature was set at 205 °C. The speed of screw rotation was set at 40 rpm. Individual extruded polymer rods were pulled and forced through a die using an air-cooled conveyor with a pulling speed in the range 90–100 rpm at room temperature to form rod-shaped filaments. The extrusion process of the blend filaments was optimized to ensure smooth surfaces of the fabricated filaments; the diameter of each extruded filament was regularly checked at every 100 cm length using a digital caliper. The average diameter of the extruded filaments was approximately 2.77 ± 0.19 mm.

### 2.3 3D Printing of the ABS/TPU Blend Filaments

The 3D printing of the ABS/TPU blend filaments was carried out using an FDM 3D printer (Ultimaker 3, Geldermalsen, The Netherlands) with a glass printing bed, as depicted in Scheme 1b. The Cura software (Ultimaker, Geldermalsen, The Netherlands) was used to export the 3D models in STL files to G-code. The optimized 3D printing parameters exploited for the extruded filaments are listed in Table 2.

**Table 2:** Summary of Fused Deposition Modeling Process Parameters for the 3D Printing of the ABS/TPU Blend Filaments

parameters	values
nozzle temperature (°C)	220–240
printing bed temperature: low, medium, high (°C)	25, 60, 80
nozzle diameter (mm)	0.8
layer thickness (mm)	0.2
raster angle	45°/–45°
print infill (%)	100

### 2.4 Characterizations of ABS/TPU Blend Filaments

#### 2.4.1 Differential Scanning Calorimetry

A differential scanning calorimeter (822e, Mettler Toledo, USA) was employed for the evaluation of transition temperatures, e.g., glass transition temperature (T_g_) and melting temperature (T_m_), of the individual ABS/TPU blend filaments. In brief, approximately 10 mg of each filament sample was placed in a sealed DSC aluminum pan. The scanning process was performed with two heating cycles (from −80 to 200 °C at a heating rate of 10 °C/min) and one intermediate cooling cycle (from 200 to −80 °C at a cooling rate of 10 °C/min). All measurements were carried out under a nitrogen atmosphere.

#### 2.4.2 Thermogravimetric Analysis

Thermogravimetric analysis (TGA, Mettler Toledo TGA/SDTA 851^e^, Switzerland) was conducted under an air atmosphere to determine the thermal stability of the prepared filaments. In brief, approximately 15 mg of a given ABS/TPU filament was loaded in a crucible pan and subsequently heated. The TGA data were collected over the temperature range of 50–700 °C at a heating rate of 20 °C/min with a gas flow rate of 40 mL/min.

#### 2.4.3 Pseudo Melt Flow Rate Measurement

To determine the proper filament printing temperature, a pseudo-melt flow rate (pseudo-MFR) measurement was performed using an FDM nozzle diameter of 0.8 mm. The molten filament material extruded through the nozzle exit at a fixed extrusion time of 30 s was weighed as a function of the printing temperature used in the range of 220–240 °C.

#### 2.4.4 Morphological Observation

The cross-sectional surfaces of the cryogenically fractured filaments were individually examined using a JEOL JSM-IT500HR scanning electron microscope (Hitachi SU3500, Horiba X-MaxN, Japan) at an accelerating voltage of 5 kV. The fractured specimens were gold-sputtered before the examination.

#### 2.4.5 Rheological Measurement

The rheological properties of all five prepared filaments, i.e., A100, A70T30, A50T50, A30T70, and T100, were examined using a parallel plate rheometer (HAAKE MARS40, Thermo Fisher Scientific, MA, USA). A dynamic frequency sweep spanning from 0.01 to 100 Hz and an oscillation strain of 1.0% were used. All measurements were performed at 225 °C using two parallel plates with a diameter of 20 mm and a gap between the plates of 1 mm. To create a sample disc, 1 g of each filament was loaded directly onto the lower plate and heated for 3 min before the upper plate was adjusted to achieve a 1 mm gap size; any excess material was carefully removed through flat trimming of the plate rim prior to the measurement. After application of the Cox-Merz rule, the complex viscosity was determined as a function of applied shear rate.

### 2.5 Characterizations of the 3D Printed ABS/TPU Blend Specimens

#### 2.5.1 Warpage Measurement

**Figure 1:**
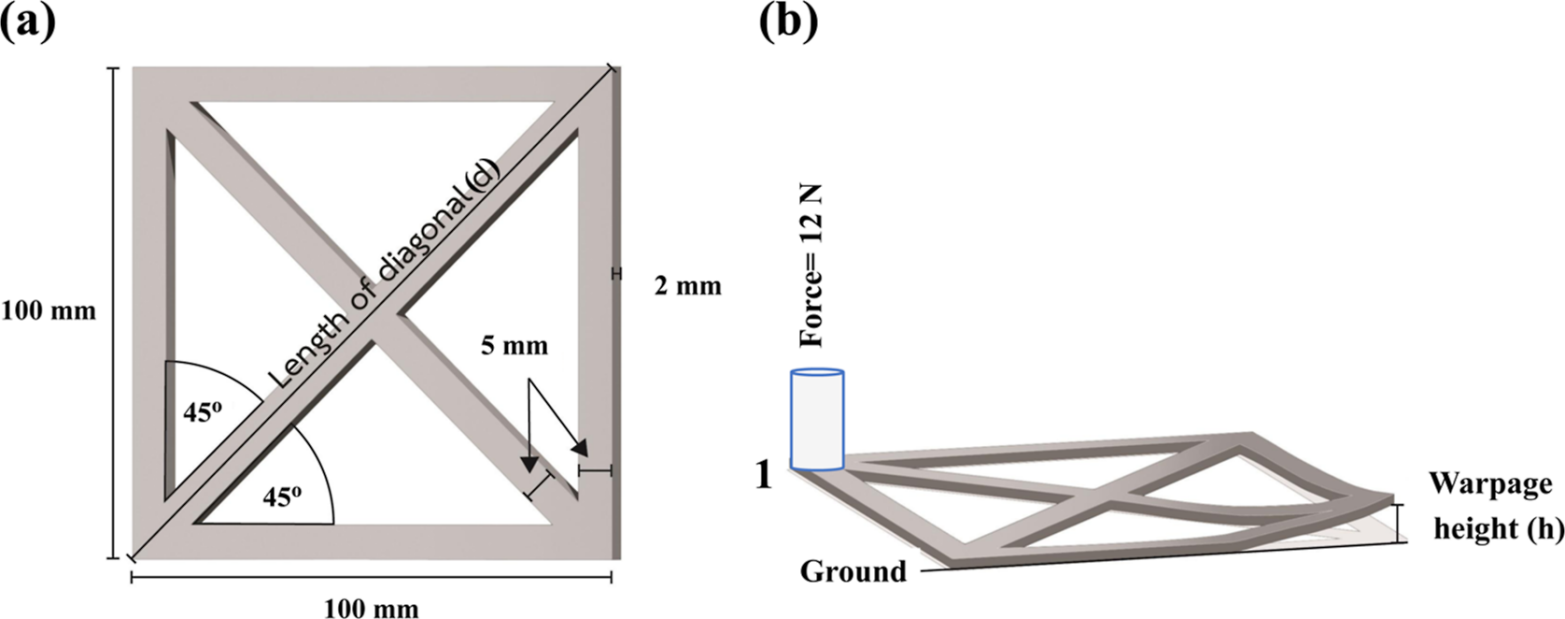
(a) CAD design of a specimen for the warpage test and (b) the setup of a test specimen for determination of a warpage height.

In general, warpage deformation occurs at the edge of a 3D printed specimen. By following the methodology proposed by Spoerk et al.,^24^ warping deformation could be promptly evaluated. Initially, the shape and dimension of an FDM-printed specimen designed for warpage analysis were created using computer-aided design (CAD) software (SolidWorks 2017, Dassault Systémes SA, USA), as shown in Figure 1a. The fabrication of the test specimens from the individual ABS/TPU filaments was subsequently conducted using the printing parameters tabulated in Table 2; no adhesive was applied on the build platform to avoid any physical specimen surface contamination. The length of the diagonal (d) of each fabricated specimen was measured, and the corner, labeled 1, of the specimen was then pressed against a constant force of 12 N for 24 h at ambient temperature before the whole specimen was photographed, as shown in Figure 1b. The warpage height (*h*) of the distorted specimen was later determined using Fiji ImageJ software (version 1.47 g). The warpage of the specimen was ultimately calculated using eq 1 listed below. The effect of build platform temperature on the warpage of a printed specimen was simultaneously investigated; three different temperatures, i.e., 25, 60, and 80 °C, were exploited.
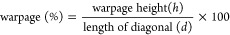
1

#### 2.5.2 Morphological Investigation

A digital microscope (Dino-Lite, AM4113/AD4113 series, Taiwan) with 50× magnification was used to examine the morphology of the deposited layers of the 3D printed dog-bone-shaped specimens with the dimensions of 5 mm width, 75 mm length, and 2 mm thickness. The finished surfaces of the selected specimens were further investigated by using a confocal laser scanning microscope (LEXT OLS4100, Olympus, Japan).

#### 2.5.3 Atomic Force Microscopy

The surface roughness (*R*_a_) and 3D topography of the FDM-printed objects were investigated by atomic force microscopy (AFM) measurements with tapping mode on an atomic force microscope (Hitachi AFM 5300E, Japan). The assessments were repeated at three defined positions (*n* = 3) of the individual specimens to determine the Ra values. The 3D topographic images (scan area of 20 × 20 μm^2^) were taken at a scan rate of 1 Hz.

### 2.6 Mechanical Property Testing

The mechanical characterization of the 3D specimens fabricated from the ABS/TPU blend filaments was carried out in different tests, i.e., tensile, flexural, and impact tests, as follows:

Tensile testing of the FDM-printed specimens (5 mm width x 75 mm length x 2 mm thickness) was performed at room temperature using a Shimadzu Universal Testing Machine equipped with a 10 kN load cell, in accordance with ISO 527.^25^ The crosshead speed was kept at 5 mm/min with a fixed gauge length of 25 mm. The results are given as the mean ± SD based on at least five separate experiments.

Flexural testing of the FDM-printed specimens (10 mm width × 80 mm length × 4 mm thickness) was conducted at room temperature on an INSTRON 55R4502 instrument using a particular crosshead testing rate of 2 mm/min with a load capacity of 10 kN, in accordance with ISO 178.^26^ All measurements were performed in at least five replicates.

Charpy impact testing of the FDM-printed specimens (10 mm width × 80 mm length × 4 mm thickness) was carried out using a Pendulum Impact Tester (GT-7045-MDH) with a 2 J pendulum hammer at an impact speed of 2.9 m/s, in accordance with ISO 179-1:2023.^27^ All measurements were performed in at least five replicates.

### 2.7 Statistical Analysis

The statistical significances for warpage analysis, surface roughness (*R*_a_) measurement, and Charpy impact test were determined using SPSS software (version 19.0; SPSS, Inc., Chicago, IL) using a one-way analysis of variance (ANOVA) with a Scheffé post-hoc test. All data were obtained from triplicate experiments unless otherwise noted and are presented as the mean ± SD. The statistical significance was set at a confidence level of 95% (*p*-value < 0.05).

## 3 Results and Discussion

### 3.1 Preparation and Properties of 3D Printable ABS/TPU Filaments

Though ABS has a high coefficient of thermal expansion, causing unfavorable shrinkage and warpage of specimens during 3D printing, it is currently still one of the most extensively exploited FDM feedstocks. To both overcome such drawbacks and develop new blend filaments with more specific and functional properties, various ABS/TPU blend filaments for FDM printing were prepared in this study via extrusion-based melt blending. The physical, thermal, and rheological properties of the extruded blend filaments, i.e., A70T30, A50T50, and A30T70, were thoroughly investigated in comparison with those of their parent polymer filaments, i.e., A100 and T100.

#### 3.1.1 Thermal Behaviors

The thermal properties of all prepared filaments were examined by DSC and are summarized in Table 3. Since both ABS and TPU are amorphous materials, the melting temperatures (T_m_) of these polymers were not observed as expected. The glass transition temperatures (T_g_) of ABS and TPU in the neat filaments were clearly detected at 103.3 °C and −31.6 °C, respectively. The T_g_ values of ABS and TPU in the blend filaments were slightly deviated; T_g_ of ABS seemed to marginally decrease with increasing TPU content, while T_g_ of TPU was not obviously altered when different amounts of ABS were blended. Nevertheless, in the presence of the least content of TPU integrated in the filament (A70T30), the T_g_ of TPU became scarcely quantified.

**Table 3:** Glass Transition Temperatures (T_g_) of ABS and TPU Present in the ABS/TPU Blend Filaments Were Determined From the DSC Second Heating Scans

	*T*_g_ of ABS [°C]		*T*_g_ of TPU [°C]	
sample code	onset	midpoint	onset	midpoint
A100	99.9	103.3		
A70T30	97.3	101.3		
A50T50	101.1	100.3	–42.0	–31.0
A30T70	93.8	100.9	–44.9	–32.8
T100			–42.6	–31.6

**Figure 2:**
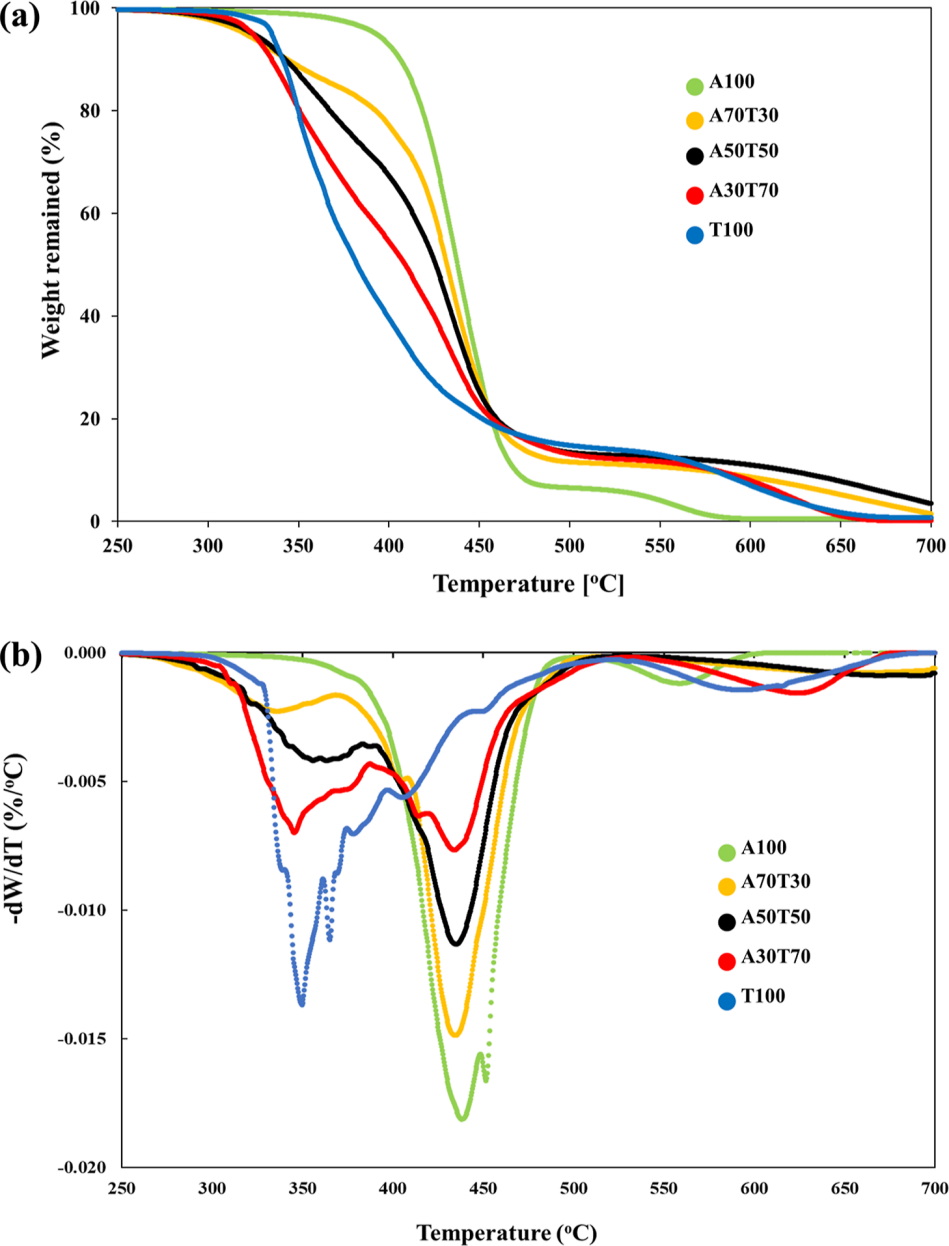
(a) TGA and (b) DTG thermograms of the ABS/TPU blend filaments being heated from 50 to 700 °C under an air atmosphere.

Thermal stability of the extruded ABS/TPU filaments was further examined by TGA; the TGA and corresponding derivative thermogravimetric analysis (DTG) curves demonstrated the changes in the mass and phase transition of each material being combusted under an air atmosphere in the temperature range of 50–700 °C (Figure 2). The thermal degradation of ABS in the neat ABS filament took place in two temperature ranges at 300–480 and 480–580 °C with a residual weight of about 0.65% at 700 °C (Table S1 in the Supporting Information). It was previously reported that the decomposition of butadiene, styrene, and acrylonitrile units of ABS began at 340, 350, and 400 °C, respectively.^28^ TPU in the neat TPU filament also degraded in two temperature ranges at 295–480 and 480–650 °C with a residual weight of approximately 0.80% at 700 °C. The first decomposition stage was associated with the scission of the urethane linkage of TPU hard segment, while the latter decomposition stage involved the degradation of the polyol chain of TPU soft segment.^29^ The acquired TGA and DTG thermograms explicitly suggested that ABS had a higher thermal stability than TPU, and both materials nearly completely burnt at 700 °C under an air atmosphere. Similar to that of the parent filaments, the thermal degradation of the blend filaments occurred in two stages, but at distinctly broader temperature ranges, as revealed in Figure 2b. The first stage was chiefly ascribed to the TPU degradation, whereas the second stage was predominantly attributed to the ABS degradation. Although the early stage of thermal degradation of the blend filaments was more sharply fainted with decreasing TPU content in the blends, the thermal degradation characteristics of the blend filaments still lie between those of the parent filaments. In addition, the residual weight percentages of the blend filaments obtained at 700 °C were overall slightly higher than those of the parent filaments, as tabulated in Table S1, plausibly suggesting a minor chemical interaction between TPU and ABS in the blend filaments, particularly those with higher contents of ABS blended.^30^ Upon being heated at elevated temperature under air, some peroxide free radicals that were generated via the oxidation of unsaturated butadiene units of ABS could possibly attack the TPU molecules, leading to slight cross-linking of the material.^31^

#### 3.1.2 Flowability

The nozzle temperature, one of the crucial FDM printing parameters, must be set properly to smoothly print a filament into a 3D object without a major structural flaw. A too low printing temperature causes insufficient material flow, while a too high printing temperature reduces material viscosity; both of which yield imperfect printed objects. According to manufacturers’ instructions, the ideal nozzle temperatures used for commercial ABS and TPU filaments are 230–250 and 200 °C, respectively; the printing temperatures of these two materials are rather different. From our preliminary study, the nozzle temperature of 250 °C was found to be too high for TPU printing, as the blackening of the printed filament was sporadically observed, which was caused by the degradation of the material under such an elevated temperature.

**Figure 3:**
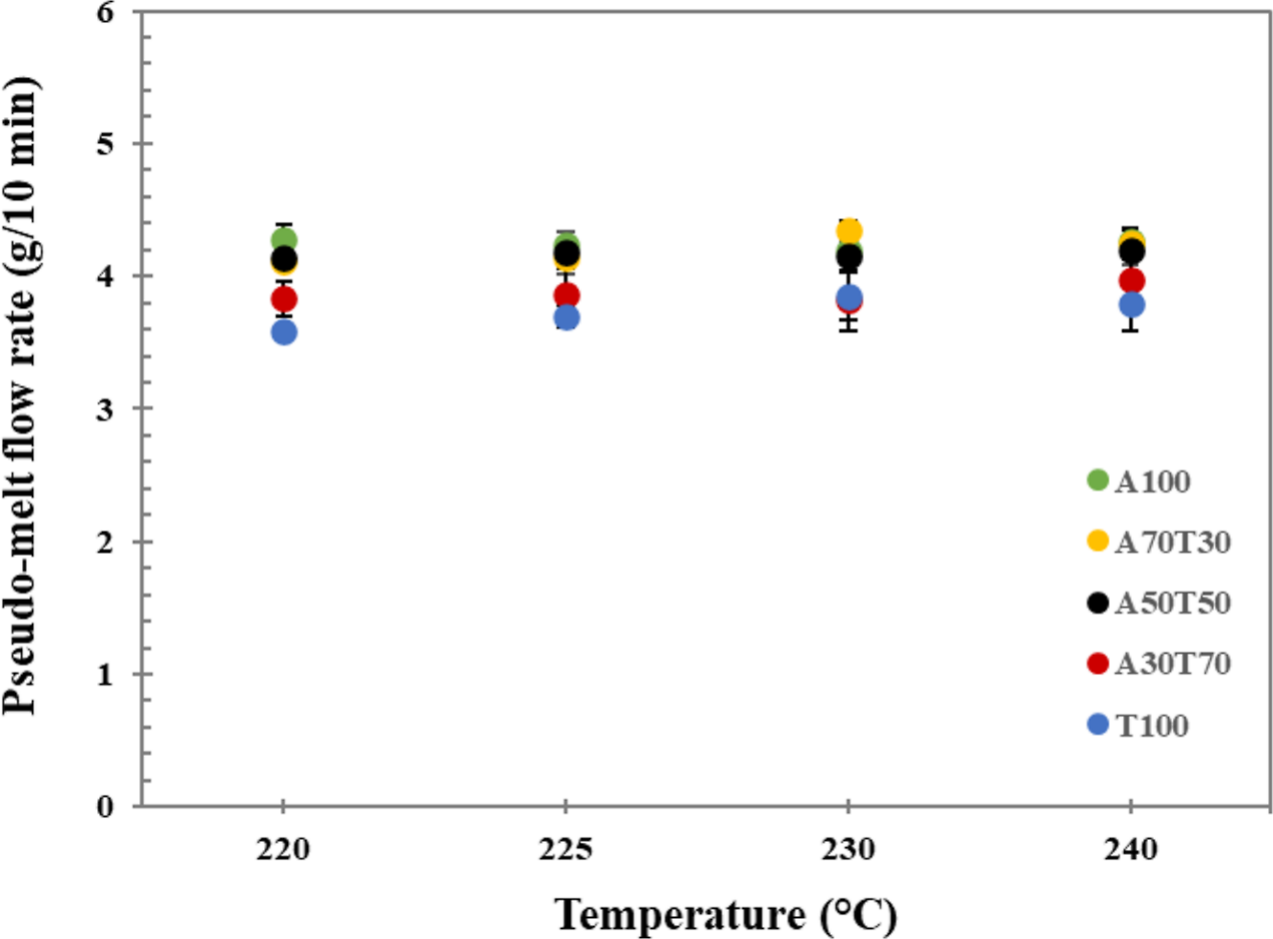
Pseudo melt flow rates of the ABS/TPU blend filaments, measured at the FDM nozzle temperatures of 220–240 °C.

The melt flow behaviors, directly related to flowability, of the blend filament were comparatively investigated with those of the corresponding neat filaments using the pseudo melt flow rate (pseudo-MFR) measurement at temperatures ranging from 220 to 240 °C. The pseudo-MFR (g/10 min) value of each test filament was directly calculated from the mass of material being extruded through the FDM nozzle for 30 s. As revealed in Figure 3, the pseudo-MFR values of the neat ABS filament appeared rather steady at the whole studied temperature range, despite the extrusion at nonrecommended nozzle temperatures below 230 °C. The pseudo-MFR value of the neat TPU filament appeared lower than that of the pure ABS filament, indicating its higher viscosity. However, the pseudo-MFR of the TPU filament slightly increased with an enhancing nozzle temperature, suggesting boosted flowability of the material. Apparently, the pseudo-MFR values of the blend filaments fell between those of their parent filaments at all temperatures studied and, overall, subsided with an increase in the content of TPU blended, as the high viscosity TPU material restricted the mobility and disturbed the flowability of the ABS chains. Even though the deviation of pseudo-MFR values of individual blend filaments, measured at 220–240 °C, was insignificant, the pseudo-MFR values of the neat and blend filaments became close to one another when the nozzle temperature of 225 °C was employed. As a result, it could be said that at this extrusion temperature, all five different ABS/TPU filaments might be most properly 3D printed.

#### 3.1.3 Morphology

**Figure 4:**
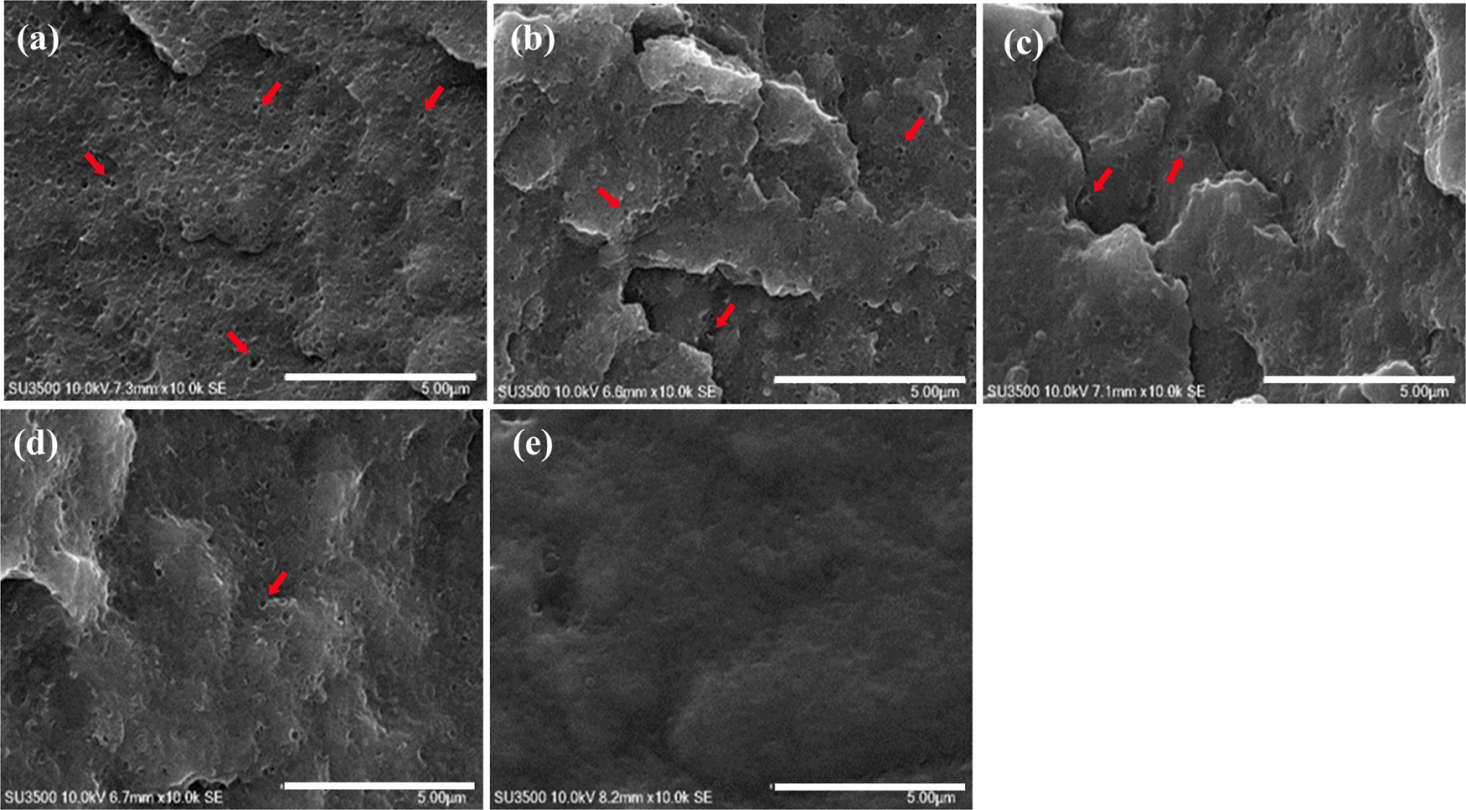
SEM images of cryofractured surfaces of the ABS/TPU blend filaments: (a) A100, (b) A70T30, (c) A50T50, (d) A30T70, and (e) T100. (Scale bar: 500 μm). Red arrows indicate small holes.

The surface morphologies of the ABS/TPU blend filaments fractured in liquid nitrogen were comparatively assessed by SEM and are shown in Figure 4. The cross-sectional surface of the pristine ABS filament (A100) revealed several tiny dens homogeneously distributed in the filament matrix (Figure 4a), which could plausibly be caused by thermal shrinkage of the material upon cooling as well as the two-phase microstructure of the material, having butadiene rubber domains grafted to styrene acrylonitrile chains. The surface of the neat TPU filament (T100) was, on the other hand, far smoother, as it possessed a relatively lower coefficient of thermal expansion (Figure 4e). Principally, ABS is hydrophobic, while TPU is hydrophilic, turning the blends of these two polymers to be thermodynamically immiscible.^32^ The number of small holes (indicated by red arrows) in the blend filament matrices seemed to noticeably decrease with an increasing amount of TPU blended, as illustrated in Figure 4b–d; they were sporadically dispersed in the filament matrices. Furthermore, the fractured surfaces of the blend filaments became smoother when a higher amount of TPU was incorporated, suggesting better compatibility of ABS and TPU, which was possibly attributed to the hydrogen bonding interactions between TPU urethane groups and ABS acrylonitrile units.^17^ Seemingly, the evenest fractured surface of the ABS-based filament was thus observed in A30T70.

#### 3.1.4 Rheological Behaviors

**Figure 5:**
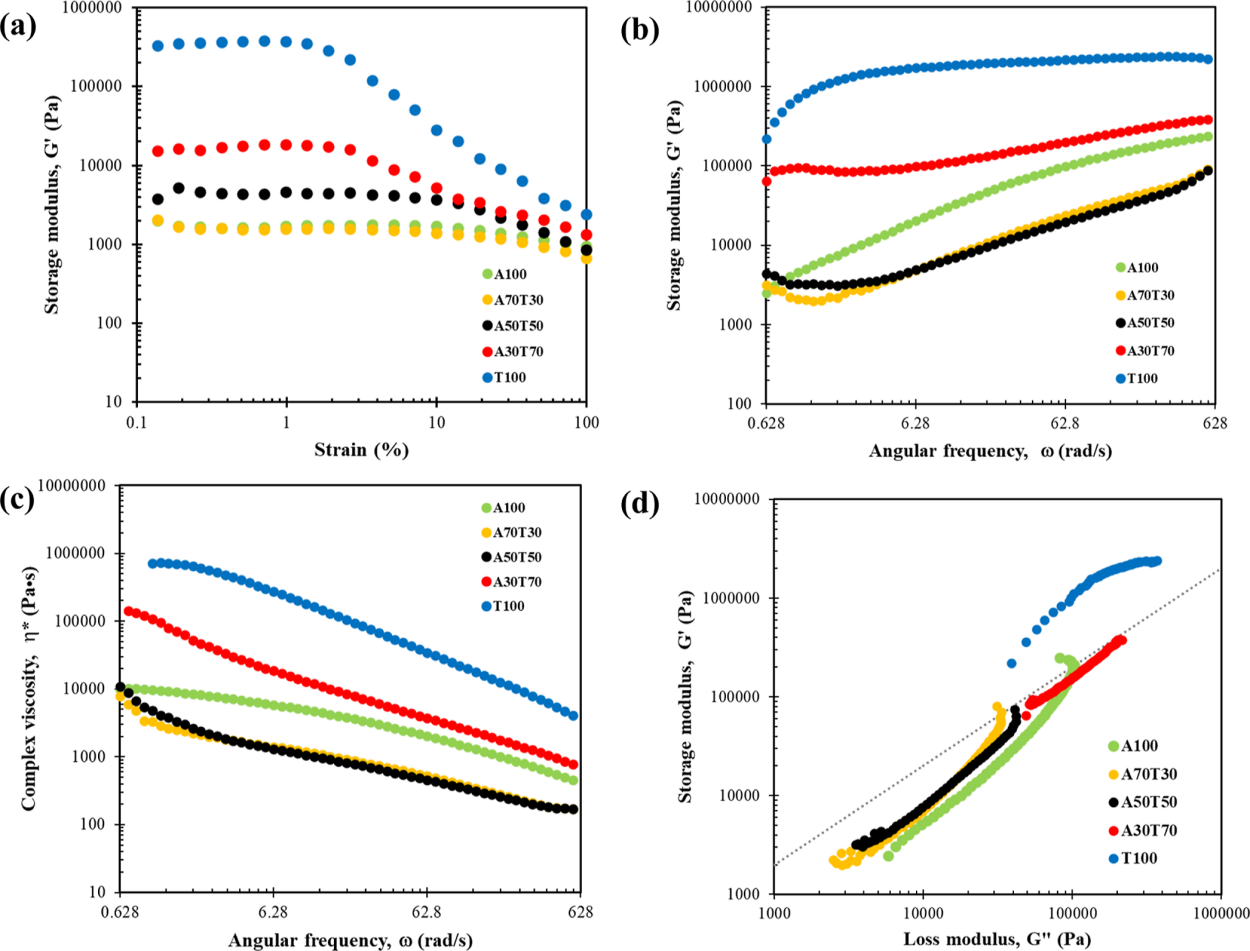
(a) Storage modulus (*G*′) as a function of strain amplitude, (b) storage modulus (*G*′) as a function of angular frequency, (c) complex viscosity (η*) as a function of angular frequency, and (d) storage modulus (*G*′) as a function of loss modulus (*G*″) of each blend specimen tested at 225 °C.

The melt viscosity of a polymer essentially plays a major role in material processability. Thus, the rheological properties of polymers are vital when the materials are to be processed. Each of the prepared ABS/TPU filaments was subsequently subjected to a dynamic strain sweep test for the determination of the linear and nonlinear viscoelastic regions. In the linear region, the material microstructure still remained stable at the shear measurement.^33^ At a critical strain value (γ_c_), an increasing strain amplitude started to disturb the material microstructure, resulting in a nonlinear viscoelastic behavior of the material. Figure 5a reveals the storage modulus (*G*′) of each filament specimen as a function of oscillatory strain amplitude value at the temperature of 225 °C. Although pure TPU material possessed the highest *G*′, it lost its linear viscoelastic behavior (Newtonian plateau) most rapidly. Hence, the higher the amount of TPU blended, the greater the *G*′ value of the specimen resulted, and the lower the γ_c_ value of the material obtained. Among the ABS/TPU test filaments, the neat TPU specimen could maintain its Newtonian flow within the shortest strain range, below 2% strain; all further rheological measurements were thus conducted in the linear regime at 1.0% strain, to ensure the linear viscoelasticity of the test materials.

Frequency sweep tests for the ABS/TPU blend specimens were conducted at a 1.0% strain amplitude over the frequency range of 0.628 to 628 rad/s (0.1 to 100 Hz). As displayed in Figure 5b, the storage modulus of each test material increased with frequency. Over the whole range of frequencies studied, the storage modulus of the pristine TPU specimen appeared greater but less susceptible to the applied frequency than that of the pure ABS specimen. At lower frequencies, the storage moduli of the neat ABS and ABS/TPU blends remained independent of frequency, likely due to the presence of butadiene regions in ABS. It is important to note that the blend composition and morphology significantly impacted the rheological behavior of ABS.^34^ The storage moduli of the ABS/TPU blends gradually increased with an increasing frequency. The addition of TPU into ABS had the potential to change the ratio between the rubber phase (butadiene) and the plastic phase (styrene-acrylonitrile) component, altering the interaction between these phases, thus influencing the rheological, morphological, and mechanical properties.^35^ Notably, all storage modulus curves of the blend samples did not fall between those of the neat polymers; only A30T70 did, suggesting that this material exhibited a well-compatible blend characteristic.^35^ Furthermore, A30T70 displayed a near plateau at low frequencies, which correlated with the TPU content of the blend. This agreed with the morphological observation of the smooth fractured surface of this blend filament which was mentioned in Section 3.1.3 above. Surprisingly, the storage moduli of the filaments blended with smaller contents of TPU, i.e., 30 and 50 wt %, became lower than that of the neat ABS filament, possibly indicating the phase-separated microstructures of such blends.^35^

Figure 5c presents the almost linear-like decrease in the complex viscosity as a function of the angular frequency of each test filament. Like the results of the relationships between storage modulus and angular frequency, the complex viscosity of A30T70 exclusively lay between those of the neat polymer filaments. Drastic reductions in the complex viscosity were, however, observed in the filaments blended with lower amounts of TPU, i.e., 30 and 50 wt %, compared with that of the pristine ABS filament, feasibly owing to the phase-separated structures of these blends. Also, by application of the Cox-Merz rule, Figure 5c can be presented as the shear viscosity. For FDM 3D printing, a relatively low shear rate is expected near the liquefier entrance and the print nozzle. Shear rates in the nozzle are commonly in the 100–200 s^–1^.^36^ From Figure 5c, the shear viscosity of all blends and the neat ABS was in the same range (around 200–2000 Pa s), which was agreeable with the melt flow index results.

Figure 5d shows a Cole–Cole plot of the storage modulus *G*′ versus the loss modulus *G*″ for the ABS/TPU blends at 225 °C. It was proposed that the log *G*′–log *G*″ should be similar if the microstructure did not alter. A slope of 2 (the dash line in Figure 5d) also indicates that the blend is miscible.^33^ The log *G*′–log *G*″ curve can also be used to elucidate structure differences of polymer materials at a fixed condition such as a fixed temperature. The increased *G*′ at a given *G*″ indicated that the microstructure of ABS changed significantly with TPU loading. Consequently, the good homogeneity of ABS and TPU in the A30T70 blend filament was confirmed by the best fitting data based on the Cole–Cole plot by means of the linear viscoelastic rheological behavior.

### 3.2 3D Printability of ABS/TPU Blend Filaments

#### 3.2.1 Warpage Analysis

**Figure 6:**
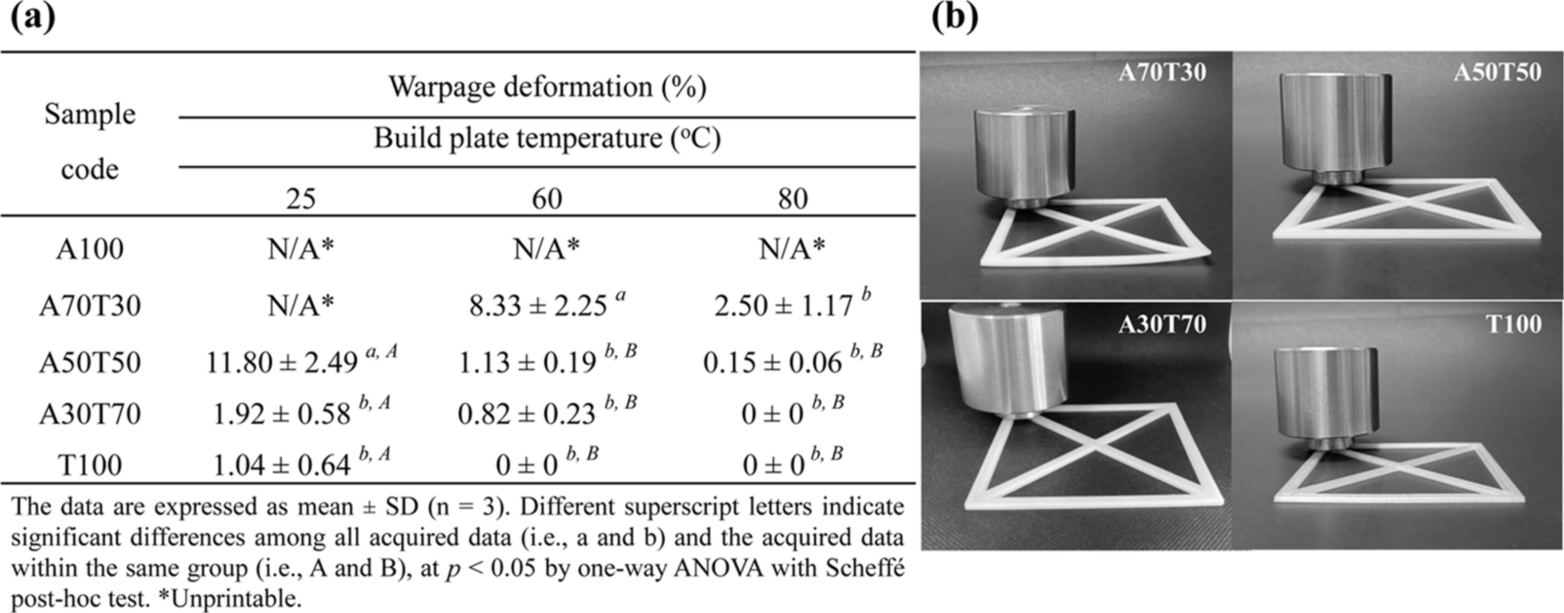
(a) Warpage deformation (%) of 3D specimens printed from the ABS/TPU blend filaments using different build plate temperatures and (b) digital photographs showing warpage deformation of the FDM-printed ABS/TPU blend specimens using a build plate temperature of 60 °C.

Warpage of 3D printed parts poses a significant challenge in FDM printing. It is essentially induced by the volumetric shrinkage of the extruded molten filament upon cooling. In this study, the 3D specimens of A100, A70T30, A50T50, A30T70, and T100 for the warpage test were printed layer-by-layer using the printing parameters stated in Table 2. The nozzle temperature was set at 225 °C, while the printing bed temperatures were set at 25, 60, and 80 °C. No adhesive or fixation spray was applied on the build platform. The specimen configuration was specifically designed by SolidWorks, as depicted in Figure 1a. The percentage of warpage of the specimen (*n* = 3) was calculated according to eq 1 (in Section 2.5.1). It was found that the pure ABS filament (A100) could not be printed into complete articles at any printing bed temperatures used. The bed temperature setting appeared too low to enable the molten material to adhere to the glass print bed. In contrast, the extruded TPU material (T100) adhered firmly to the glass print bed at all printing bed temperatures employed, owing to the presence of highly polar components and hydrogen bonds formed in the hard segment of TPU. As illustrated in Figure 6a, not only was the printability of the ABS-based filaments enhanced, but also the warpages of the 3D printed objects were reduced when increasing amounts of TPU were blended in the filaments. As anticipated, the specimens printed from A30T70 at all printing bed temperatures had more significantly decreased warpage (%), compared to those fabricated from A70T30 and A50T50 at the same printing bed temperatures. This was primarily attributed to the relatively lower coefficient of thermal expansion of TPU compared to that of ABS. Nevertheless, the most well-blended matrix of A30T70, derived mostly from the intermolecular force, especially hydrogen bonding, between ABS and TPU, also played a major role in the enhancement of printability and reduction of warpage deformation. Generally, the strength of intermolecular interaction, known as compatibility between two polymers in the blend, reaches homogeneity (miscibility) in strong intermolecular bonding.^37^

The influence of printing bed temperature on warping behavior was also systematically examined in the context of 3D-printed specimens with varying weight ratios of ABS to TPU. As illustrated in Figure 6a, increasing printing bed temperature was found to have a pronounced effect on mitigating warpage deformation as it not only assisted the adhesion of the printed layers but substantially enhanced the adhesive strength of the TPU interface bonding within the blends, which was consistent with the previously reported interfacial adhesion properties of TPU.^15^Figure 6b displays digital photographs of different 3D ABS/TPU specimens printed with a build plate temperature of 60 °C. The entire T100 specimen lay completely flat on the bench without warpage deformation, which slightly differed from the specimen printed with the unheated glass printing bed; warpage of 1.04% was obtained despite the high polar nature and the low coefficient of thermal expansion of TPU. Taken together, both TPU blending content and printing bed temperature had direct effects on the alleviation of warpage deformation in the FDM-printed ABS/TPU blend specimens.

#### 3.2.2 Morphology Observations

**Figure 7:**
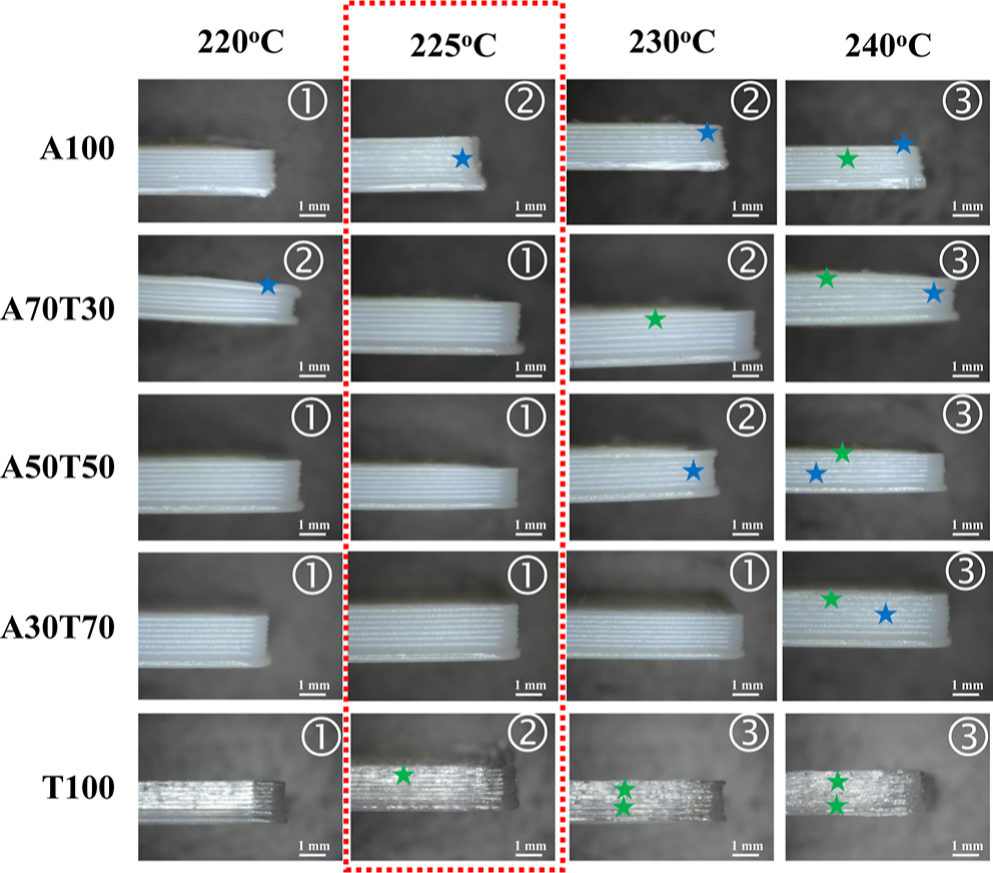
Stereoscopic images of side views of 3D ABS/TPU blend specimens printed at different printing temperatures with a use of a printing bed temperature of 60 °C. 1, 2, and 3 are designated for good-, medium- and poor-surface qualities of the deposited layers of the individual specimens, respectively. Blue and green stars denote the defects generated by nonhomogeneous flows and overflows of printing filaments, respectively. Scale bar = 1 mm.

The surface morphologies of the deposited layers of the FDM-printed dog-bone shaped ABS/TPU blend specimens, with a dimension of 5 mm width, 75 mm length, and 2 mm thickness, were first examined by a digital microscope with a 50 times magnification. The observations were focused on the side views (deposited layers) of the individual specimens (*n* = 2), which were printed layer-by-layer using the printing parameters listed in Table 2 at various printing temperatures, i.e., 220, 225, 230 and 240 °C, and printing bed temperature of 60 °C. The surface quality of each sample was arbitrarily graded in three distinct levels: 1 = good quality, 2 = medium quality, and 3 = poor quality. In addition, visual inspections were performed across the interlayer regions of the specimens; defects caused by nonhomogeneous flows and overflows of filament materials were indicated by blue and green stars, respectively. To be more specific, the specimens displayed uniformly deposited layers and smooth interlayer regions throughout the whole specimen, indicating good quality (level 1). The surface quality of the medium grade (level 2) exhibited minimal defects resulting from either nonhomogeneous flows or overflows of filament materials. The poor grade (level 3) had more defects arising from both nonhomogeneous flows and overflows of filament materials or a high number of filament material overflows. As shown in Figure 7, though it is generally recommended to print a commercial ABS filament at 230–250 °C, most of the FDM-printed ABS specimens exhibited well-defined deposited layers, except those fabricated at 240 °C. Meanwhile, the surfaces of the fabricated TPU objects tended to swell and overflow from the bottom to top layers printed at temperatures above 220 °C, where too high fluidity or too low viscosity of the extruded TPU feedstock readily occurred, indicating that TPU was more temperature-sensitive than ABS.

Like the 3D TPU specimen, the fabricated blend specimens still exhibited the overflow depositions of the filaments extruded at high printing temperatures, but this time, it happened at temperatures above 225 °C. However, the most suitable printing temperature to obtain the homogeneous flows of the filaments was exclusively 225 °C, the A70T30 object printed at 220 °C still possessed some uneven depositions of the extruded filament, particularly at the edges and vertices. This observation could be attributed to the deficient compatibility of the two polymers blended at a low TPU content, which totally agreed with the results of morphological observation of the fractured surface by SEM as well as the rheological property of A70T30 mentioned above. Nevertheless, all printing defects observed between the stacked layers of all 3D printed ABS/TPU specimens were not severe.

**Figure 8:**
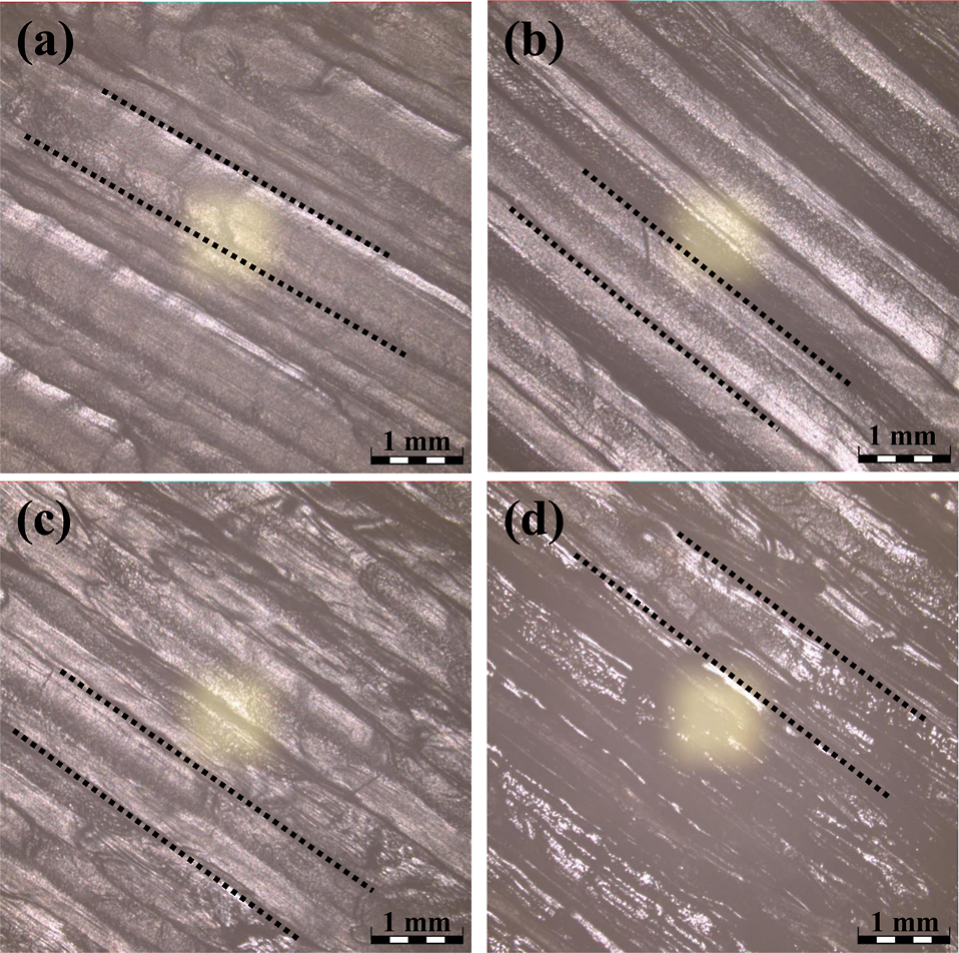
Confocal laser scanning microscopic images of the A30T70 specimens fabricated at the printing temperatures of (a) 220 °C, (b) 225 °C, (c) 230 °C, and (d) 240 °C. Scale bar = 1 mm.

The surface morphology of the dog-bone shaped A30T70 specimens printed at 220, 225, 230, and 240 °C was further examined by confocal laser scanning microscopy (CLSM). The CLSM image of the specimen fabricated at 220 °C displayed a linear, but irregular, surface pattern, as seen in Figure 8. This was plausibly derived from the imperfect melting of the filament blended with a large amount of high-viscosity TPU material while being extruded through the FDM nozzle. A more uniform and consistent linear printing structure was observed on the surface of the specimen printed at a higher temperature, i.e., 225 °C, suggesting a more proper printing temperature, allowing a better melting and flowability of the extruded material. However, the fabrication of A30T70 specimens using excessive heat at temperatures above 225 °C triggered overflow of the printing material, resulting in surface flaws. Taken together with the warpage results, the 3D printing of the ABS/TPU filaments for the subsequent studies was conducted with a nozzle temperature of 225 °C and a printing bed temperature of 60 °C.

**Figure 9:**
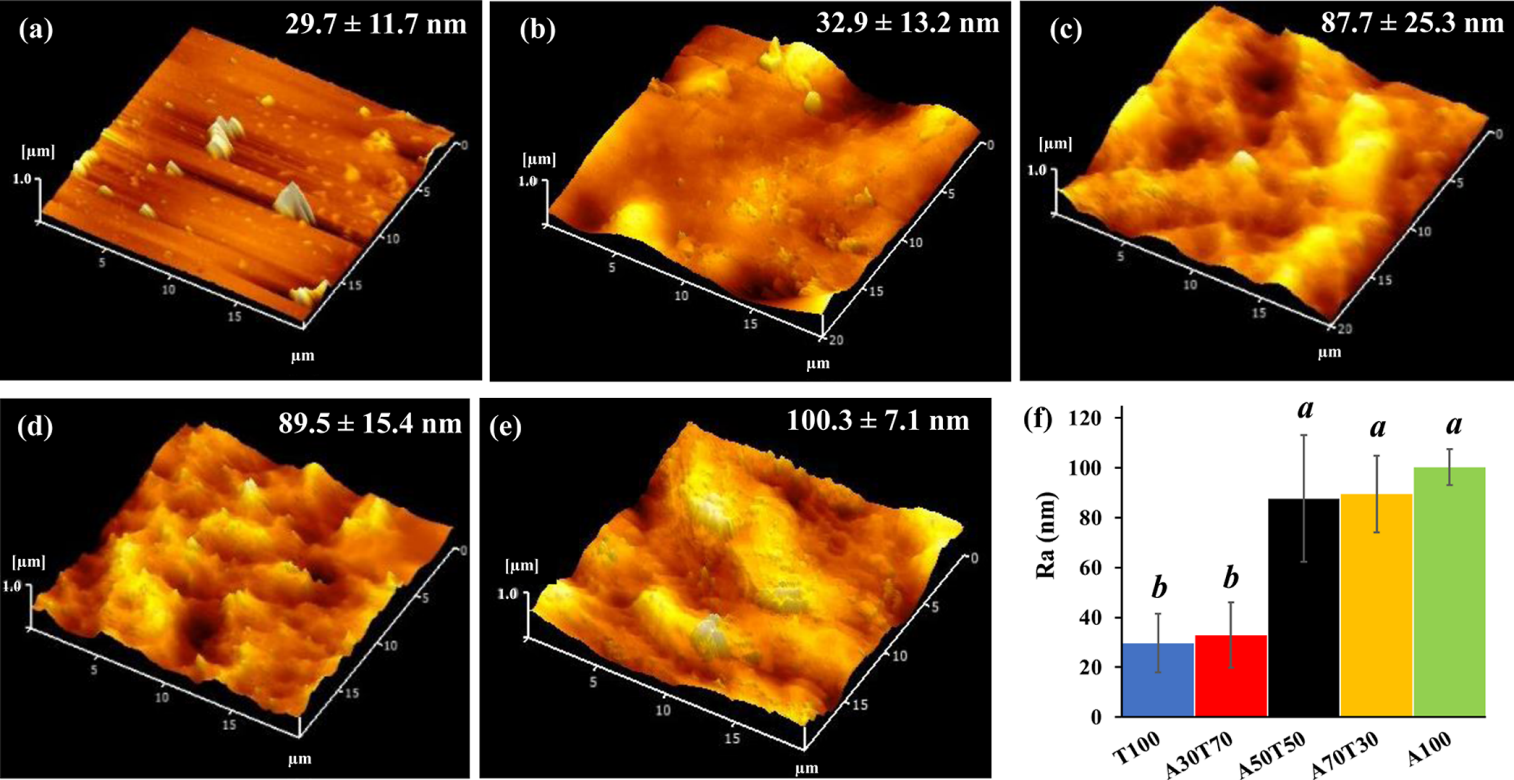
AFM phase images of the 3D specimens fabricated from the different filaments: (a) T100, (b) A30T70, (c) A50T50, (d) A70T30, and (e) A100 (*X*- and *Y*-axes are presented in 20 μm whereas *Z* axis is presented in 1 μm.) and (f) the plot of surface roughness (*R*_a_) values of the 3D specimens, expressed as mean ± SD (*n* = 3) [Different letters (a and b) indicate significant differences at *p* < 0.05, analyzed by one-way ANOVA with Scheffe post-hoc test.].

The individual disk specimens with 20 mm diameter and 2 mm thickness were prepared from the pristine TPU and blend filaments for the assessment of surface roughness by atomic force microscopy (AFM) on 20 μm × 20 μm scan areas (*n* = 3). The AFM phase images of the 3D ABS/TPU blend objects are comparatively shown with their average surface roughness (*R*_a_) values in Figure 9. The surface morphology of the pure TPU specimen displayed the dark brown and smooth region (Figure 9a), similar to that previously observed,^38^ while the light brown and rough region corresponded to the pure ABS specimen (Figure 9e).^17^ When ABS was blended in the ABS/TPU filaments, it was imaged as a light gold texture region, similar to that disclosed in the literature.^39^ Not only was the surface roughness of the 3D specimens drastically enlarged but also the homogeneity of the blends was somewhat decreased with an increasing amount of ABS blended (Figure 9b–d). Among the 3D blend objects, the fabricated A30T70 specimen exhibited the smoothest surface morphology (Figure 9d) with the lowest surface roughness value of 32.9 ± 13.2 nm, which was rather close to that of the printed T100 specimen. This was unquestionably associated with the good homogeneity of the blend filament prepared at this ABS-to-TPU weight ratio as well as the most proper printing parameters, particularly both the printing temperature and the printing bed temperature.

### 3.3 Mechanical Performances of 3D Printed ABS/TPU Blend Specimens

**Figure 10:**
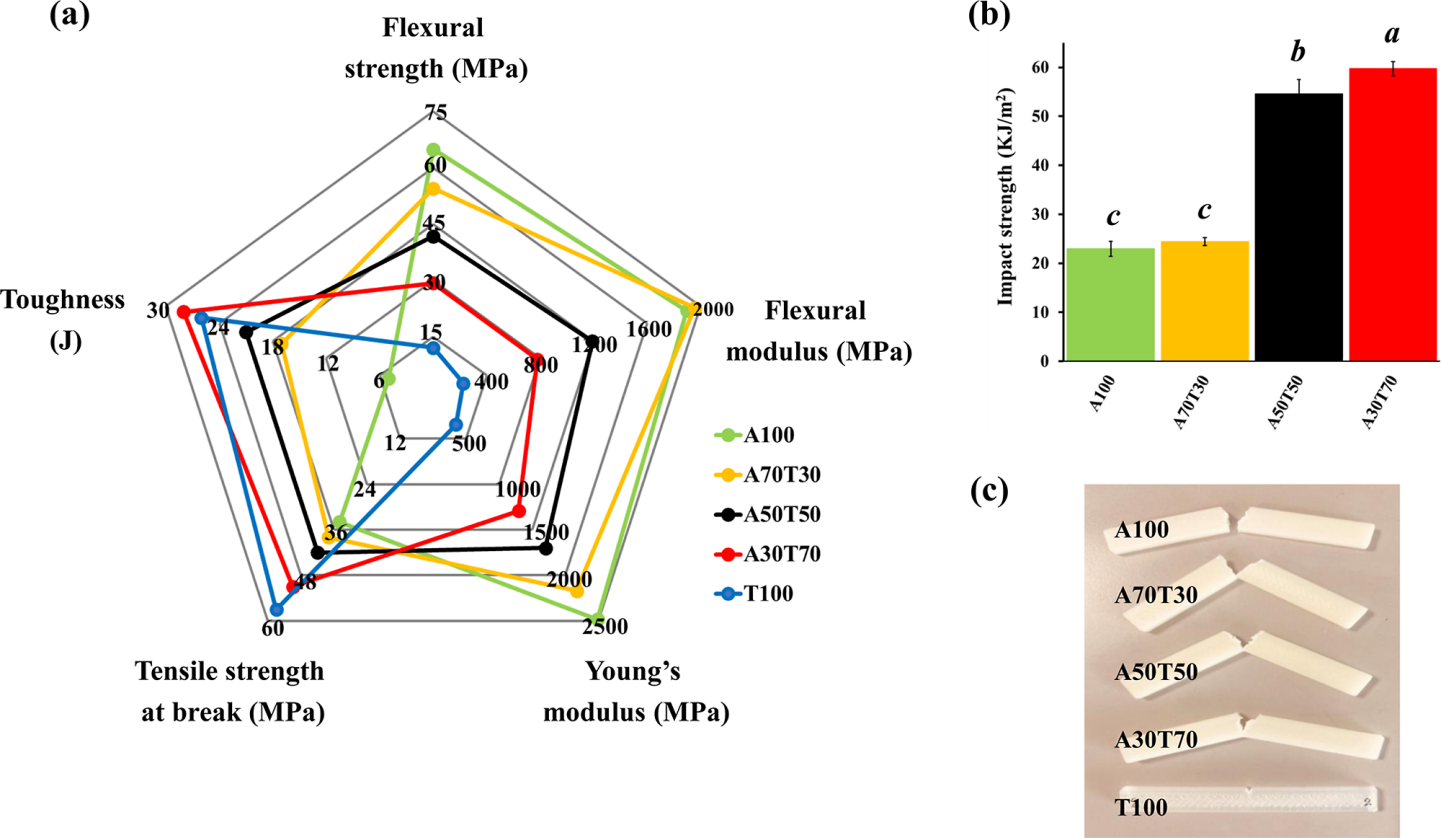
(a) A radar chart summarizing the mechanical properties of the FDM-printed specimens, (b) Charpy notched impact strength of the FDM-printed specimens (The data are expressed as mean ± SD (*n* = 5), and the different letters (a, b, and c) indicate significant differences at *p* < 0.05, analyzed by one-way ANOVA with Scheffé post-hoc test.), and (c) the images of the fractured specimens captured after Charpy V-notch test.

The mechanical properties of 3D printed specimens (*XY* orientation) from the ABS/TPU blend filaments were evaluated by tensile, flexural, and impact. The representative radar chart, illustrated in Figure 10a, displays the tensile and flexural performances of the FDM-printed specimens. As revealed in Figure S1 (in the Supporting Information), the printed T100 sample was weakest and toughest, while the fabricated A100 object was strongest and stiffest (most readily broken). It was evident that the increasing TPU content in the blends led to decreased Young’s moduli and enhanced tensile strength at break and toughness of the FDM-printed specimens. By blending varied amounts of TPU in the filaments, both tensile strength at break and toughness of the 3D specimens were gradually increased from 34 ± 1 MPa and 5 ± 0.5 J (for pure ABS object) up to 51 ± 1 MPa and 28 ± 3 J (for A30T70 object), respectively. The noticeably heightened elongation of the blend samples was absolutely associated with the TPU component. Consequently, the Young’s moduli of the 3D objects subsided considerably about 48%, from 2484 ± 59 MPa (for A100 article) to 1293 ± 34 MPa (for A30T70 article). Furthermore, as expected, the tensile strength of the 3D printed neat ABS object was found to be lower than that of the pure ABS object fabricated by a conventional process (as indicated in Table 1). This was unquestionable because the fabrication of 3D-printed objects involves a layer-by-layer deposition formation, which exhibits anisotropic characteristic and drawbacks such as poor interlayer adhesion and incomplete printing, resulting in lowered mechanical performance and structural failure.^40^

The flexural strength and modulus of each printed specimen are displayed in Figures 10a and S2. The incorporation of TPU into the filaments markedly cut down both strength and modulus values of the materials to about half, i.e., 57 and 59%, respectively (from those of the 3D A100 specimen to those of the 3D A30T70 specimen) as the flexibility of the 3D blend samples was substantially increased by the presence of the TPU component.

Charpy impact strength (impact energy) of the FDM-printed samples was determined as a function of the amount of TPU blended. It was noted that the notched TPU sample did not break under the test conditions because it was too tough; the impact strength (resilience) of the 3D-printed TPU specimen could not be, consequently, measured. Noticeably, acting as an impact modifier, TPU assisted the 3D blend objects in absorbing more energy. Hence, the resilience of the printed material boosted notably from 22.95 ± 1.49 (for A100 article) to 59.72 ± 1.43 kJ/m^2^ (for A30T70 article). This was in accordance with the enhanced toughness of the printed blend specimens obtained by the tensile test. Seemingly, the impact strength of the fabricated A70T30 sample was insignificantly increased compared to that of the A100 object. The integration of 30% by weight of TPU into the blend filament scarcely improved the resilience of the blend material; this was possibly attributed to the imperfect blending of this material and the testing position of the specimen.^41^Figure 10c illustrates the different fracture characteristics of the test specimens. The printed ABS sample was totally torn apart, while the fabricated A70T30 specimen was nearly completely broken, indicating that the latter material was relatively tougher. The A30T70 fracture exhibited the shortest crack formulation and propagation in comparison to those of the other blend samples, denoting its greatest ductility. Nevertheless, the overall impact strength of the fabricated blend specimens was progressively intensified with an increasing content of TPU, indicating that this polymer served as a good and effective toughener for ABS.

## 4 Conclusions

In the present study, ABS/TPU blend filaments for 3D printing were prepared via a melt-mixing method. The properties and printability of the extruded blend filaments were directly governed by the blend composition. The most suitable nozzle temperature exploited for 3D printing of all blend filaments was found to be 225 °C. Together with the surface morphology observation by SEM, a Cole–Cole plot by means of the linear viscoelastic rheological behavior indicated the good homogeneity of ABS and TPU in A30T70 despite the thermodynamically immiscible blending of these two polymers. Consequently, this assured the high quality of 3D-printed specimens in terms of good layer adhesion and less warping while printing. In addition, the substantially improved surface roughness of the A30T70 object resulted with the usage of the optimized printing parameters, particularly the nozzle and printing bed temperatures. Though the strength and modulus values of the fabricated blend materials were reduced with the increasing amount of TPU blended, the printed articles could absorb more energy, as TPU helped toughen the entire materials. The whole findings suggested that the well-tuned blend composition and printing parameters of the ABS/TPU filaments enabled A30T70 to be a promising FDM filament feedstock.

## Data Availability

Data will be made available on request.

## Supporting Information Available

Residual weight percentages of the ABS/TPU blend filaments after being combusted under air atmosphere, tensile and flexural stress–strain curves, and flexural properties of the 3D specimens printed from the different ABS/TPU blend filaments (PDF)
