# Supplementary material for: Acrylonitrile Butadiene Styrene/Thermoplastic Polyurethane Blends for Material Extrusion Three-Dimensional Printing: Effects of Blend Composition on Printability and Properties
Source: ACS Omega. 2023 Nov 17;8(47):45013–25. doi: 10.1021/acsomega.3c06595 (PMC10688206; doi:10.1021/acsomega.3c06595)
Supplement: Supplementary file 1 — ao3c06595_si_001.pdf [file ao3c06595_si_001.pdf]

Supporting Information

Acrylonitrile Butadiene Styrene/Thermoplastic Polyurethane Blends for Material Extrusion Three-Dimensional Printing: Effects of Blend Composition on Printability and Properties

Boonlom Thavornyutikarn[1,a], Chuanchom Aumnate[2,a], Wasana Kosorn[1], Nutdanai Nampichai[1], Wanida Janvikul[1,*]

[1]*National Metal and Materials Technology Center, National Science and Technology Development Agency, Pathum Thani, Thailand*

[2]*Metallurgy and Materials Science Research Institute, Chulalongkorn University, Bangkok, Thailand*

[a] These authors equally contributed to this work.

*Correspondence: Wanida Janvikul, E-mail: wanidaj@mtec.or.th

Table S1. The residual weight percentages of the ABS/TPU blend filaments after being combusted under air atmosphere in the temperature range of 50-700°C, determined by TGA.

Sample code	Residue at 700°C (wt%)
A100	0.65
A70T30	1.62
A50T50	3.62
A30T70	0.32
T100	0.80

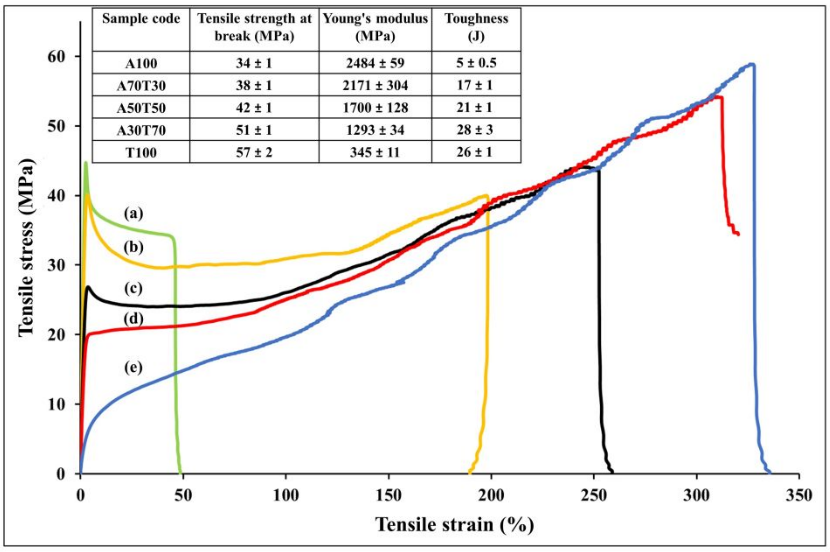

Figure S1. The tensile stress-strain curves and tensile properties of the 3D specimens printed from the different ABS/TPU blend filaments: (a) A100, (b) A70T30, (c) A50T50, (d) A30T70, and (e) T100.

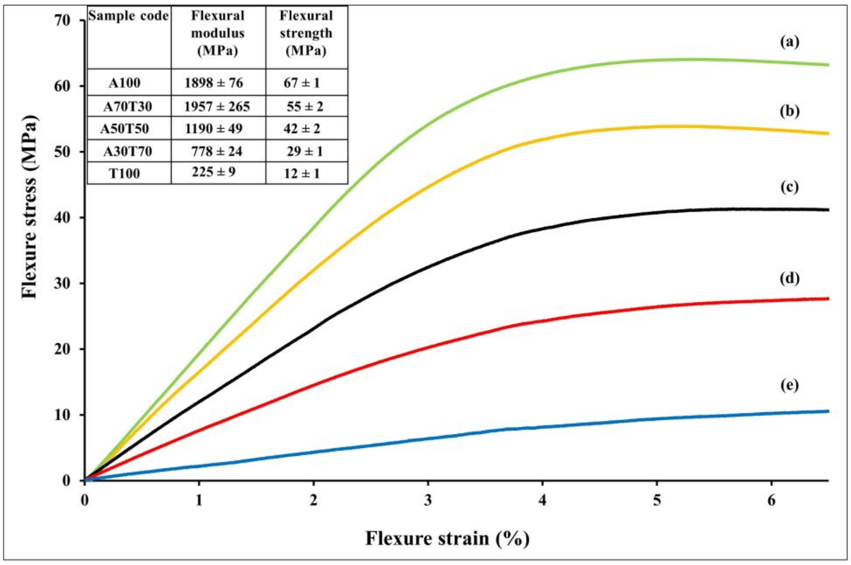

Figure S2. The flexural stress-strain curves and flexural properties of the 3D specimens printed from the different ABS/TPU blend filaments: (a) A100, (b) A70T30, (c) A50T50, (d) A30T70, and (e) T100.
